# Examining preservice mathematics teachers’ perceptions of technological pedagogical content knowledge (TPACK) pathways: a structural equation modeling approach

**DOI:** 10.3389/fpsyg.2026.1929322

**Published:** 2026-10-08

**Authors:** Yan-hui Xu

**Affiliations:** College of Mathematics and Physics, Wenzhou University, Wenzhou, Zhejiang, China

**Keywords:** exploratory factor analysis, preservice mathematics teachers, structural equation modeling, teacher professional development, TPACK

## Abstract

**Introduction:**

When TPACK is first proposed, it is believed to consist of 7 components, and 3 of them are core components: TK, PK and CK; another 4 components are compound ones overlapping the 3 core components, which are: TPK, TCK, PCK and TPACK. Many subsequent empirical studies find validity of the TPACK framework has not always been supported. This study adopts structural equation modeling to accurately measure and construct the TPACK structure perceived by preservice mathematics teachers, and based on this, proposes several strategies of promoting the formation and development of preservice teachers’ TPACK structure.

**Methods:**

To investigate the internal structure and developmental pathways of their TPACK, this study surveyed 368 preservice mathematics teachers from a local comprehensive university in China by using an adapted PMT-TPACK questionnaire. By employing exploratory factor analysis (EFA) and confirmatory factor analysis (CFA), a four-dimensional TPACK model was constructed and validated.

**Results and conclusions:**

The model consists of content knowledge (CK), technological knowledge (TK), technological content knowledge (TCK), and integrated TPACK. The results reveal that preservice mathematics teachers can’t distinguish the boundaries among technological pedagogical knowledge (TPK), pedagogical content knowledge (PCK) and synthesized TPACK. This study has also found that preservice teachers have the strongest confidence in TCK. The confidence in TK is significantly low, especially in terms of self-evaluation in learning new technologies and using professional software. The findings indicate that TCK demonstrates a significant mediating effect between CK and integrated TPACK, the level of participation in technical courses and educational practice courses has a significant weak positive impact on all dimensions of TPACK. This suggests that the development of TPACK is an integrative process driven by both coursework and practical experience, with TCK acting as a critical mediator. Based on these conclusions, it is recommended that teacher education programs should systematically develop a curriculum that deeply integrates technology, content, and pedagogy, reinforce the central role of integrative knowledge, and enhance preservice teachers’ ability of integrating technology into effective teaching practice through progressive, hands-on experiences.

## Introduction

Based on the framework of pedagogical content knowledge (PCK) as proposed by Shulman, Koehler and Mishra (2005) proposed the concept of technological pedagogical content knowledge (TPACK), which offers a clear framework for teachers’ professional knowledge in the digital era. By mastering TPACK, teachers can better understand and apply technology, which helps them effectively integrate technology into subject teaching, improve teaching quality, and enhance student learning experience. TPACK consists of 7 components, and 3 of them are core components: technology knowledge (TK), pedagogical knowledge (PK) and content knowledge (CK); another 4 components are compound ones overlapping the 3 core components, which are: technological pedagogical knowledge (TPK), technological content knowledge (TCK), pedagogical content knowledge (PCK) and technological pedagogical content knowledge (TPACK) (Koehler and Mishra, 2005).

Mishra and Koehler (2006) pointed out that in teaching practice, the TPACK framework describes the kinds of knowledge that teachers need in order to teach with ICT, and the complex ways in which these bodies of knowledge interact with one another rather than being separated. TPACK is a conceptual framework for understanding how technology can be integrated effectively into classrooms (Koehler and Mishra, 2009). The TPACK framework has received significant attention from educators and researchers. About 600 publications on TPACK were indexed in the Scopus database from 2011 to 2019 (Tseng et al., 2022). However, different scholars hold different views on what these concepts represent or how to define them. Especially in different disciplines, the components of TPACK and their relationships may vary. Mathematics is a highly abstract and logically rigorous discipline. In the case of mathematics education, the teacher must not only know mathematics and pedagogy, but also how to teach this discipline. Proper use of technology means in mathematics teaching can effectively promote concept understanding and interest of learning. Integrating technology in mathematics lessons may assist teachers in creating engaging and lively classes (Mhlongo et al., 2023), which may support students in overcoming learning barriers through practical experiences instead of simple rote learning (Shambare and Simuja, 2022).

However, previous studies have found that preservice mathematics teachers do not equip necessary ICT skills which are needed in practice to integrating ICT into mathematics teaching (Wang, 2009). Jiang et al. (2020) pointed out that although preservice teachers are generally familiar with digital tools and have high technological acceptance, they still face significant difficulties in organically integrating technology, pedagogy, and abstract mathematical content, often resulting in the phenomenon of “using technology for the sake of technology”. Preservice mathematics teachers generally face the dilemma of “knowing but not doing” or “acting but being not efficient” in teaching practice of integrating technology. Although preservice mathematics teachers have mastered basic technical operation skills and mathematical subject knowledge, it is often difficult for them to integrate technology when teaching mathematics. Based on the above background and issues, the constructs of technology, pedagogy and content affect TPACK independently (Koehler and Mishra, 2005; Niess, 2005). Effective teaching with technology also depends highly on these knowledge domains (Harris et al., 2009).

This study takes preservice mathematics teachers from college of mathematics and physics at a local comprehensive university in China as the research subjects. Utilizing the questionnaire survey method, combined with exploratory factor analysis and structural equation modeling, this study explores: firstly, the core constituent dimensions of TPACK perceived by preservice mathematics teachers. Secondly, the reliability and validity of the TPACK questionnaire. Thirdly, the structural relationships among the dimensions of the TPACK questionnaire. Fourthly, the influence path of demographic variables on TPACK development. Through empirical analysis, this study aims to construct and validate a structural model of TPACK perceived by preservice mathematics teachers, and provide empirical evidence for developing preservice mathematics teachers’ TPACK.

## Theoretical framework

### The TPACK framework

Since the initial publication of TPACK in 2006, it is becoming a popular analytic lens for studying teacher knowledge about educational technologies and has been used in a number of published research articles (e.g., Hofer and Swan, 2008; Koehler et al., 2007; Özgün-Koca et al., 2010; Syh-Jong and Kuan-Chung, 2010). Pierson (2001) described TPCK as the multi domain knowledge and skills that teachers possess in specific grades and subject situations. Angeli and Valanides (2005) presented PCK related to ICT. Hughes (2004), Wallace (2004), Margerum-Leys and Marx (2002), Margerum-Leys (2004) have shown sensitivity and attention to the relationship between content, pedagogy, and technology. Niess (2005) rejected Pierson's (2001) definition of TPCK, stating that it is a strategic way of thinking that encompasses not only knowledge and skills, but also developing subject content knowledge, technological knowledge, and pedagogical knowledge. It is a dynamic concept. Niess (2005) proposed a framework for describing the development outcomes of TPACK based on Grossman’s (1991, 1990, 1989) work, stating that TPACK has four core elements: (1) a holistic approach to integrating technology into specific subject areas (such as mathematics) teaching; (2) Using technology to support specific thematic teaching strategies and representation of knowledge, such as mathematics; (3) Knowledge about students’ understanding, thinking, and learning with the support of technology in specific disciplines; (4) Integrate knowledge of technology and course materials (such as mathematics) learning, as well as knowledge related to the course. Niess proposed the four core elements of TPACK, which comprehensively consider the teacher’s concept and the knowledge required for teachers to integrate technology into teaching, learning, and curriculum.

Mishra and Koehler (2006) aimed to systematically explain how teachers can effectively integrate technology into subject teaching. This theory proposed by Mishra & Koehler quickly became the core model for studying teachers’ ability of integrating technology due to its emphasis on the relationship and integration among technology, pedagogy, and content. Niess (2006) pointed out that it is comprehensive effect of different knowledge components that supports teachers’ use of technology in teaching. Niess does not aim for TPACK, but rather uses the synthesis of three types of knowledge as a way of supporting technological teaching. Koehler et al. (2007) argue that the development of TPACK is a “multigenerational process.” Hofer and Swan (2008) examined the practical TPACK, arguing that TPACK is a dynamic construct. Each teacher possesses his/her own knowledge of content, pedagogy, and technology, and TPACK even varies depending on a teacher’s context across different scenarios. Koehler and Mishra (2008) argue that using technology for teaching is within a certain context, which endows teachers with the flexibility they need to succeed. Teachers need to have a deep understanding of the interactions among three types of knowledge and how they intertwine in specific contexts. Therefore, the environment pulse has become an indispensable part of the TPACK framework.

Angeli and Valanides (2009) drew inspiration from Cochran et al’s PCKg model, placing the development of teachers’ pedagogical and content knowledge within the context of their understanding of learners and learning environments, and limiting the technology in Koehler & Mishra model to ICT, proposing the concept of ICT-TPCK. Cox and Graham (2009) argue that TPACK is a “sliding” framework based on existing technology. When the functionality of a technology becomes transparent and most teachers use it, the knowledge foundation for using this technology for teaching is no longer TPACK, but PCK. When the knowledge represented by technology becomes universally available, TCK becomes CK. When the importance of technology is no longer emphasized, TPK becomes PK. The framework proposed by Cox and Graham (2009) has a certain effect on distinguishing various knowledge components in TPACK, and the recognition of TPACK’s “sliding features” also helps better understand its relationship with PCK.

Wassong and Biehler (2010) proposed a model for teacher knowledge and competence. An important feature of this model is to represent various knowledge components in the form of three concentric circles. Among them, the outermost circle consists of three core knowledge components -TK, PK, and CK, among which CK is divided into common content knowledge (CCK), special content knowledge (SCK) and horizon knowledge (HK). The middle circle is divided into technological content knowledge (TCK), technological pedagogical knowledge (TPK), knowledge of content and teaching (KCT), knowledge of curriculum (KC), knowledge of content and student (KCS). The innermost layer is TPCK. The unique feature of this model is the integration of two conceptual frameworks of teacher knowledge, represented by concentric circles, demonstrating the unique role of technology in teacher knowledge in a layered and in-depth manner. Guerrero (2010) refined the components of TPACK based on the characteristics of mathematics, dividing it into four parts: conception and use of technology, technology-based mathematics instruction, technology-based classroom management, depth and breadth of content.

Graham (2011) examined the TPACK framework according to the criteria for establishing theory proposed by Whetten (1989) and pointed out that: (1) From the perspective of “what is it”, TPACK framework has three shortcomings: ① TPACK is built on an uncertain concept (PCK). What exactly is PCK? There is no unified definition in the academic community, and different researchers have different understandings of it. TPACK is built on the basis of PCK, what is destined to be a difficult concept to define precisely. ② The contradiction between the formal simplicity and internal complexity of TPACK framework. TPACK framework succinctly represents the relationship among technology, content, and pedagogy through three circles and their intersections. However, behind this form of simplicity lies potential internal complexity. As Mishra and Koehler (2006) stated, TPACK has “complex, multifaceted, and contextual characteristics” (p.1017). ③ TPACK framework lacks precise definitions for each element, and different researchers have different definitions for the same element. For example, according to Cox's (2008) research, there are 89 different definitions of TPACK in various literature, which makes this concept ambiguous. Another example is that Mishra and Koehler (2006) and Koehler and Mishra (2008, 2009) have provided different definitions of technology.

(2) From the perspective of “how,” TPACK framework also has at least two shortcomings. ① TPACK framework has not effectively addressed the issue of whether various knowledge components are integrated or transformed. The “integrated view” holds that TPACK is a mixture of various knowledge components. The “transformational view” holds that TPACK is a new form of knowledge that cannot be explained by the sum of its parts. From the Venn diagram of the TPACK framework, it appears that TPACK is formed by the integration of three types of knowledge: technological knowledge, pedagogical knowledge, and content knowledge. However, according to Mishra and Koehler (2006) and Koehler and Mishra (2008, 2009), TPACK is “transformed” from the aforementioned three types of knowledge. ② The boundaries among the various knowledge components of TPACK framework are not clear, such as the differences among TPK, TCK, and TPACK, which are sometimes difficult to distinguish.

(3) From the perspective of ‘why’, researchers have not yet fully demonstrated the value of TPACK framework. The researchers did not specify the value of each element in TPACK framework. Although Mishra & Koehler have provided some clarification on the value of TPACK framework, for example, they argue that a common criticism of current educational technology is that it is “used for the sake of technology, not for teaching purposes,” TPACK framework provides us with a common language to discuss the existing or missing connections in the conceptualization process of educational technology (Mishra and Koehler, 2006, p.1044). However, TPACK framework involves three core elements and four composite elements, and the value of their existence has not been well addressed in current research on TPACK framework.

Despite the considerable ambiguity and inconsistency in the definition of TPACK and its subcomponents, there is a considerable body of research in the literature that adopted the TPACK framework (Koehler and Mishra, 2008) and sought to find methodologically sound ways to measure TPCK in terms of its subcomponents (Chai et al., 2011b; Harris and Hofer, 2011; Schmidt et al., 2009). The empirical findings from this line of research have not always been supportive of the validity of the TPACK framework (Angeli and Valanides, 2009, 2013; Archambault and Barnett, 2010; Archambault and Crippen, 2009; Graham, 2011; Shinas et al., 2013). Indicatively, it is mentioned here that Archambault and Barnett’s (2010) study revealed that the highly accepted seven mutually exclusive domains of the TPACK framework may not exist in practice, since it is difficult to distinguish each of the knowledge domains.

### Understanding TPACK pathways through structural equation modeling

Mishra and Koehler’s (2006) TPACK framework proposes two pathways to TPACK. The first is through the direct influences of TK, PK, and CK whereas the second is through the effects of the intermediary knowledge forms of TPK, TCK, and PCK. But the two TPACK pathways proposed in the TPACK framework have not been expressly investigated in these qualitative studies. While there is some evidence speaking to the existence of TPACK pathways, it is unclear if the effects of TK, PK, and CK are as strong as the effects from the intermediary knowledge forms of TCK, TPK, and PCK (Koh et al., 2013). Many qualitative studies have described examples of the seven TPACK constructs for particular subject areas and argued that these different forms of knowledge do exist (e.g., Özgün-Koca et al., 2010). Some found evidence of TPACK emerging as teachers made connections between their TK, PK, and CK (Koehler and Mishra, 2005; Koehler et al., 2007) but did not attempt to describe the specific influence of each (Cox and Graham, 2009).

A considerable body of research on the assessment of TPACK was mainly conducted through surveys, interviews, observations, and performance assessments. Among these approaches surveys were the most reported research instrument (Koehler et al., 2012). The most widely used survey instrument is the questionnaire developed by Schmidt et al. (2009) for preservice teachers. Schmidt et al. (2009) found that in a sample of preservice teachers in the United States, TPK dimension scored the highest, TK and PCK were relatively low, and TPK, TCK, and PCK had the strongest correlation with TPACK. This result lays the foundation for subsequent quantitative research. Several subsequent studies modified Schmidt et al.’s survey to examine pre-service teachers’ TPACK (Chai et al., 2010; Chai et al., 2011a, 2011b; Chai et al., 2013; Dong et al., 2015; Koh et al., 2010; Xiong et al., 2020). However, as research is conducted in different cultural contexts, it has been found that the structure of TPACK is not always stable and consistent. For example, Chai et al. (2010)‘s results indicated that CK, PK, and TK positively influenced these teachers’ TPACK, with PK having the greatest effect. The participants’ perception of TK was the lowest in both pre-and post-ICT training. But preservice teachers generally have difficulty distinguishing TCK, PCK, and TPK. Koh et al. (2010)‘s analyses yielded five distinctive constructs: CK, TK, KP (knowledge of pedagogy), KTT (knowledge of teaching with technology), and KCR (knowledge from critical reflection). The participants could not distinguish between TPK, TCK, and TPACK, and the items of these constructs were loaded as KTT. Koh et al. (2010) found that teachers, perceived TPACK to be formulated from the direct effects of technological knowledge and pedagogical knowledge. They also perceived these knowledge sources to contribute to the development of technological pedagogical knowledge and technological content knowledge, which also contributed to their TPACK. In these teachers’ conceptions of TPACK, however, the effects of content knowledge and pedagogical content knowledge were not evident.

Lee and Tsai (2010) also developed a TPACK survey to assess Taiwanese teachers’ web-based TPACK that was based on the constructs of TK, TCK, TPK, and TPACK. Their exploratory factor analysis of survey responses also found the items of TCK and TPK loading as a factor. In addition, Lee and Tsai were able to obtain good fit indexes for their four-factor model through confirmatory factor analysis. When examining the interactions among TPACK structures, Chai et al. (2011b) only extracted five factors, excluding TCK and PCK. Among them, the items of TPK, TCK, and TPACK were merged into a single dimension of “technological pedagogical knowledge”, indicating that participants had difficulty distinguishing these three types of highly integrated knowledge clearly. Chai et al. (2011b) also used a stepwise regression model and found that PK and TPK have the strongest impact on the preservice teachers’ TPACK, but only TPK and TCK are important indicators of TPACK in the TPACK structure. Shinas et al. (2013) revealed that participants did not always make conceptual distinctions between the TPACK domains. Specifically, factors were congruent across only technological knowledge (TK) and content knowledge (CK) and not congruent across pedagogical knowledge (PK), pedagogical content knowledge (PCK), technological content knowledge (TCK), and TPACK. Additionally, PK and PCK loaded together, indicating the participants did not distinguish PK from PCK. Shinas et al. (2013) confirms the need to provide more clarity about the TPACK framework and to revisit survey instruments built directly around the framework.

Zhang et al. (2015) investigated the structural and quantitative relationships among variables in the preservice teachers’ TPACK structure. The research results indicate that in the preservice teachers’ TPACK structure, single element knowledge has a positive impact on subject pedagogical knowledge that integrates technology through composite element knowledge. Among them, the pedagogical knowledge that integrates technology has the most significant impact, while the impact of technological knowledge is the least significant. The confirmatory factor analysis conducted by Wei and Zhou (2015) and Xu et al. (2018) on Chinese preservice teachers showed that although the dimensions were highly correlated, they could still be effectively distinguished structurally, and TCK and TPK had strong predictive effects on TPACK. Valtonen et al. (2017) examine the challenges presented by the psychometric properties of existing TPACK survey instruments, then share a new, validated questionnaire that focuses upon preservice teachers’ self-reported TPACK and 21st century skills. Valtonen et al. (2017) suggest that such work can “outline the effects that the TPACK components have on one another, as well as identify the weak and strong areas that require special consideration in teacher education.

There are several studies indicating a greater or lesser number of factors than seven: Archambault and Barnett (2010) reported the existence of three factors, Zelkowski et al. (2013) reached a four-factor structure. The studies by Koh et al. (2010) and Chai et al. (2011b) revealed five factors. In all these studies some of the original factors were merged together. In addition, it was found that pre-service teachers did not link CK to TPACK (Chai et al., 2011b). Pre-service teachers could not clearly perceive the differences between TCK, PCK, and TPK (Qiu et al., 2022). These research differences indicate that the structure of TPACK is not universally fixed, but is influenced by factors such as cultural background, disciplinary characteristics, and learner’s development stage, and its internal dimensional relationships have obvious contextual dependence. The development of TPACK will also be influenced by multiple factors such as individual characteristics and external cultivation environment. At the individual level, Zhan (2011) found that male preservice teachers typically exhibit stronger confidence in technical operation related dimensions, while female preservice teachers have advantages in teaching design and classroom organization. In terms of training paths, the design of the curriculum system has a direct impact on the development of TPACK. Zhang et al. (2016) found through comparing different teaching modes that the curriculum form of “case teaching combined with practical reflection” is the most effective in promoting preservice teachers to truly integrate technology into teaching thinking, and its effect is significantly better than simply teaching or skill training.

In addition, various influencing factors do not exist in isolation, but are intertwined and work together. Zhao et al. (2018) pointed out that educational internship experience can significantly improve the learning effectiveness of technical courses. Preservice teachers who have actually used technical tools during internships have significantly higher TPACK levels than their peers who only receive classroom training. Njiku et al. (2021) found lesson design has been significant in improving TPACK among pre-service teachers and it has gained more recognition in initial teacher education. Similarly, Nguyen et al. (2022) also emphasise the role of collaborative and practical tasks in promoting the development of TPACK among pre-service teachers. Xiong et al. (2020) investigated the perceived TPACK of 807 Chinese pre-service teachers. Their results yielded all seven constructs. The study participants rated CK and TK the lowest. Qiu et al. (2022) investigated the TPACK factor structure of 286 pre-service TCSL teachers via exploratory factor analysis, which yielded a six-factor structure. The results revealed that the teachers could not distinguish the boundaries between technological pedagogical knowledge (TPK) and technological content knowledge (TCK); and TPK and synthesized TPACK. Further, confirmatory factor analysis using structural equation modeling substantiated the validity and reliability of the adapted 32-item TCSL-TPACK survey instrument. Qiu et al. (2022) also found that the pre-service teachers were slightly satisfied with their overall TPACK but were least confident of their technological knowledge (TK), and the more experienced pre-service teachers exhibited higher confidence in all six factors.

Many of these studies highlight the key value of the combination of learning and application, indicating that theoretical learning is difficult to transform into effective integration ability without the support of real teaching contexts. Angula and Simuja (2025) pointed out that the role of context within the TPACK model is crucial yet often overlooked. Scholars interpret context differently. Aspects such as subject matter, student level, learners’ backgrounds, and available technology can all define context. Also, the structure and attributes of the learning environment can shape context. Other considerations like the classroom setup, the school’s resources, and external factors such as state and national standards may also define context. Understanding these variables may enhance our grasp of how TPACK is formed and adjusted to various teaching and learning situations (Nepembe and Simuja, 2023). Therefore, the development of preservice teachers’ TPACK is essentially a dynamic process gradually constructed through the interaction of multiple factors, requiring systematic and interconnected training support. Due to the different performances of TPACK in different disciplines, researchers need to delve into specific disciplines for in-depth research (Hsueh, 2008). Survey items should be contextualized with context -specific pedagogy and technology (Qiu et al., 2022).

### Research questions

Although TPACK theory provides a theoretical basis for the study of teacher’s knowledge structure in the information age, due to its complexity and ambiguity, there is a lack of accurate description of the relationships among various types of knowledge in teacher’s TPACK structure. Many studies show TPACK to be a complex theoretical framework still needing much development and validation (Cox and Graham, 2009). Methodologically, researchers still face many challenges when attempting to formulate structural equation models for the TPACK framework. Few published TPACK surveys have been evaluated for their construct validity (Koh et al., 2013). Therefore, most of the related research remains at the theoretical level, and the conclusions obtained have certain shortcomings in solving the problem of training teacher’s TPACK in practice. In response to the above issues, this study adopts structural equation modeling to accurately measure and construct the TPACK structure perceived by preservice teachers, and based on this, proposes several strategies of promoting the formation and development of preservice teachers’ TPACK structure. Based on the literature reviewed above, the current study would like to investigate the following questions: What are the main dimensions of TPACK perceived by preservice mathematics teachers? What is the overall structure roughly like? What is the correlation among these dimensions? Which components may play a more critical role in the formation of integrated TPACK? Will background factors such as gender and grade level, as well as learning experience in educational practice and technology related courses, have an impact on the preservice mathematics teachers’ TPACK level?

## Methodology

### Instrument development study

The survey tool used in the study was adapted based on existing mature scales. The questionnaire items mainly referred to the TPACK measurement questionnaire developed by Chai et al. (2010) and Koh and Divaharan (2011), and were appropriately adjusted and optimized in combination with the characteristics of mathematics and the preservice mathematics teachers’ actual situation. In the early stage of questionnaire development, the researcher invited two mathematics education research experts to evaluate the surface validity and content validity of the questionnaire, and also solicited opinions from five mathematics education master students on the rationality of question design and the standardization of wording. Based on feedback, the researcher revised and streamlined the initial items, ultimately obtained a survey questionnaire (PMT-TPACK) consisting of 33 items. The distribution of items in each dimension includes 6 questions on technological knowledge (TK), 5 questions on pedagogical knowledge (PK), 4 questions on content knowledge (CK), 4 questions on pedagogical content knowledge (PCK), 5 questions on technological content knowledge (TCK), 5 questions on technological pedagogical knowledge (TPK), and 4 questions on integrated technological pedagogical content knowledge (TPACK). All items in this survey questionnaire are scored according to the seven point Likert scale (1 = Strong disagree; 2 = disagree; 3 = something disagree; 4 = uncertain; 5 = something agree; 6 = agree; 7 = strong agree.). Please refer to the appendix for specific survey items.

### Participants

This study surveyed preservice mathematics teachers from the school of mathematics and physics at a local comprehensive university in Zhejiang Province in China, covering four grades: freshman, sophomore, junior, and master’s graduate students. A total of nine classes were involved, and 368 valid questionnaires were collected. The sample consists of 52 freshman students, 137 sophomore students, 153 junior students, and 27 graduate students. Among them, there are 182 males and 186 females, with a basically balanced gender distribution. We conducted a questionnaire survey in groups based on the class. The entire questionnaire should be filled out within approximately 10 min, using the break time between classes for participants to complete the questionnaire. The questionnaire was collected on-site by the researcher. The recovery rate is 98%. To ensure the privacy of participants and the anonymity of the research, the survey did not collect personal identity information such as names. Only four demographic variables, including grade, gender, participation in educational practice courses, and participation in educational technology courses, were recorded for subsequent analysis.

### Data analysis

The study used SPSS 23.0 software and borrowed the research method of Field and Golubitsky (2009) to conduct exploratory factor analysis on 33 items by using the maximum variance rotation method. Subsequently, the factor structure extracted from EFA was subjected to confirmatory factor analysis (CFA) by using AMOS 28.0 software, and the analysis process employed maximum likelihood estimation.

## Results

### Exploratory factor analysis and descriptive statistics

To test whether the sample size is sufficient and the correlation among the questionnaire items, SPSS software was used for KMO test and Bartlett’s test. The test results showed a KMO value of 0.93, Bartlett sphericity test χ^2^ = 2879.30 (df = 231), *p* < 0.000, indicating that the sample data meets the requirements of factor analysis.

Exploratory factor analysis adopts a “step-by-step exploration” method and follows the following principles to extract factors: (1) Delete 6 items with factor loadings<0.5 (delete items: TK4, TCK4, PK4, PCK1, PK2, PK5); (2) Delete a total of 3 cross load items (Delete items: PK1, PK3, TCK5); (3) Delete 2 items with common factor variance<0.5 (Delete items: TPACK2, TK6). As shown in Table 1, the final result retained 22 valid items, extracted 4 common factors, and explained 68.5% of the total variance cumulatively (≥60% is considered good), indicating that common factors can effectively reflect the original item information. According to the significance of each item in each factor, it can be seen that factor 1 reflects the participants’ mastery of basic mathematics knowledge, which is named content knowledge (CK). Factor 2 reflects the participants’ confidence in software technology and other proficiency, which is named technological knowledge (TK). Factor 3 mainly reflects the participants’ ability of integrating mathematical content knowledge and technological tools, which is named technological content knowledge (TCK). Factor 4 reflects the participants’ comprehensive teaching ability of integrating technology, content, and pedagogy, and is named technological pedagogical content knowledge (TPACK).

**Table 1:** Factor loadings, *α* coefficients, and mean values of the PMT-TPACK questionnaire.

Item	Factor 1	Factor 2	Factor 3	Factor 4	Mean	SD
*CK*, *α = 0.85*					*5.16*	*0.93*
CK1	0.77				5.06	1.14
CK2	0.73				5.39	1.18
CK3	0.58				5.25	1.06
CK4	0.75				4.94	1.13
*TK*, *α = 0.85*					*5.15*	*1.13*
TK1		0.66			4.94	1.25
TK2		0.90			5.25	1.33
TK3		0.86			5.27	1.26
*TCK*, *α = 0.87*					*5.44*	*0.89*
TCK1			0.69		5.28	1.18
TCK2			0.82		5.39	1.06
TCK3			0.68		5.27	1.08
TK5			0.69		5.64	1.04
TPK1			0.64		5.62	1.10
*TPACK*, *α = 0.94*					*5.13*	*0.86*
TPACK1				0.60	5.08	1.09
TPACK3				0.52	5.19	1.02
TPACK4				0.63	5.13	1.07
TPK2				0.67	5.18	1.12
TPK3				0.76	5.15	1.09
TPK4				0.69	5.11	1.10
TPK5				0.67	5.19	1.10
PCK2				0.66	5.13	1.07
PCK3				0.80	5.05	1.13
PCK4				0.73	5.13	1.05

Reliability is an indicator for testing reliability and stability. In this study, Cronbach’s *α* coefficient was used to test the internal consistency reliability of the questionnaire. The results showed that *α* coefficient of the overall questionnaire was 0.951, and *α* coefficient of each factor was as follows: CK (*α* = 0.85), TK (*α* = 0.85), TCK (*α* = 0.87), TPACK (*α* = 0.94) (see Table 1 for details). The values of *α* coefficient of the overall questionnaire and each factor are all greater than 0.8, meeting the reliability requirements for social science research and confirming the reliability of the questionnaire.

To gain a preliminary understanding of the basic situation of the sample, descriptive statistics will be conducted on the questionnaire data first. As shown in Table 1, the overall confidence level of preservice mathematics teachers is above average: the average score of TPACK is 5.13, with a variance of 0.86, which is highly consistent with the anchor point (5 points) of “somewhat agree” in the scale, indicating that preservice mathematics teachers have a certain level of confidence in their integrating technology into PCK. The average scores of the other three subscales range from 5.1 to 5.5, with specific distributions shown in Table 1. In terms of various factors, their confidence levels also show significant differences, as analyzed in detail as follows.

The highest score was obtained for TCK (*M* = 5.44, SD = 0.89), indicating that pre service mathematics teachers are confident in their core ability of using technology to support mathematics teaching. Specifically, participants showed outstanding performance in multiple technology adapted mathematics teaching questions: “Using basic office applications (such as PPT)” (TK5: *M* = 5.64; SD = 1.04) ranked among the top. The confidence in using appropriate technology (such as electronic whiteboards) to present mathematics teaching content (TCK2: *M* = 5.39; SD = 1.06) and using software specifically designed for mathematics teaching or learning (such as Geometry Sketchpad) (TCK1: *M* = 5.28; SD = 1.18) is also strong. At the same time, the self-assessment score of “using internet search engines (such as Baidu) to prepare teaching materials” (TPK1: *M* = 5.62; SD = 1.10) was also high.

Technical knowledge (TK) is one of the dimensions with relatively low scores (*M* = 5.15; SD = 1.13), which is a confidence weakness for preservice mathematics teachers. Among them, “learning new technologies” (TK1: *M* = 4.94; SD = 1.25) has the lowest self-assessment score. The confidence in using basic image software (such as Meitu Xiuxiu) (TK2: *M* = 5.25; SD = 1.33) and using basic video software (such as Jianying) (TK3: *M* = 5.27; SD = 1.26) is slightly higher than TK1, but still does not reach the average level of TCK dimension.

CK’s score is similar to TK’s (CK: *M* = 5.16; SD = 0.93). In this dimension, the three items of “having sufficient understanding of mathematical knowledge (such as mathematical concepts)” (CK1: *M* = 5.06; SD = 1.14), “having confidence in becoming a mathematics teacher” (CK2: *M* = 5.39; SD = 1.18), and “using multiple ways of deepening understanding of mathematical knowledge” (CK3: *M* = 5.25; SD = 1.06) have an average score of over 5 points, reflecting the preservice mathematics teachers’ confidence in their own mathematical subject foundation, teaching professional competence, and the ability of deepening mathematics knowledge. However, the score of “keeping up with the latest developments in mathematics teaching and thinking about related issues” (CK4: *M* = 4.94; SD = 1.13) is below 5 points, indicating that the preservice mathematics teachers’ ability of perceiving themselves dynamically at the forefront of mathematics teaching is relatively insufficient.

To further explore the intrinsic correlation between various factors of TPACK, Pearson correlation analysis was conducted on data from four subscales. As shown in Table 2, all factors were significantly positively correlated at the *p* < 0.01 level, with correlation coefficients ranging from 0.47 to 0.74. Among them, TCK has the highest correlation with TPACK (*r* = 0.74), followed by CK and TPACK (*r* = 0.69). The significant positive correlation among various factors confirms the internal logical consistency of the TPACK framework, that is, the integration of technological knowledge, content knowledge, and pedagogical knowledge is the core of TPACK formation, and also indicates that the measurement dimensions of this study have good correlation validity.

**Table 2:** PMT-TPACK correlation analysis.

Correlation coefficient	CK	TK	TCK	TPACK
CK	1.00			
TK	0.47^**^	1.00		
TCK	0.64^**^	0.53^**^	1.00	
TPACK	0.69^**^	0.54^**^	0.74^**^	1.00

^**^p < 0.01.

### Confirmatory factor analysis

Through exploratory factor analysis, four common factors were identified for TPACK perceived by preservice mathematics teachers. Based on this, the study referred to Mishra and Koehler's (2006) TPACK framework and constructed a modified hypothesis model (see Figure 1). Assuming the following statement: H1: CK has a significant positive effect on TCK. H2: CK has a significant positive effect on TPACK. H3: TK has a significant positive effect on TCK. H4: TK has a significant positive impact on TPACK. H5: TCK has a significant positive impact on TPACK.

**Figure 1:**
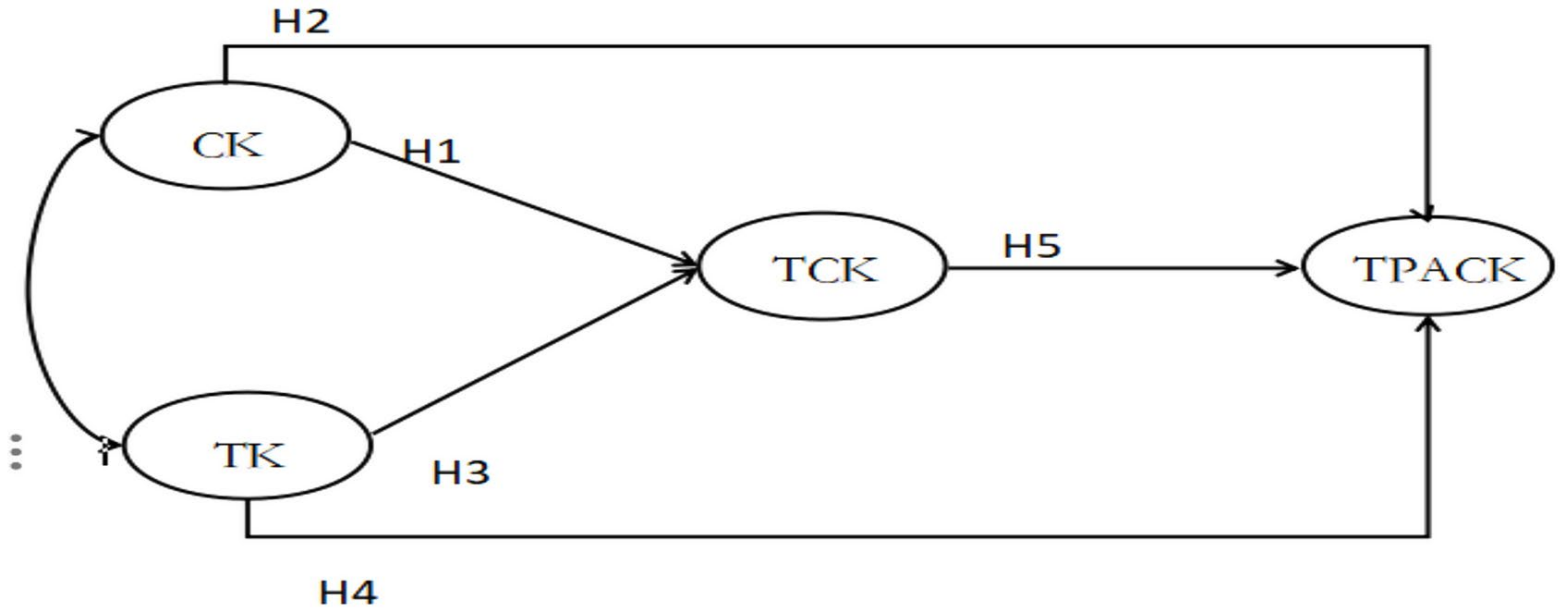
The assumed structural model of PMT-TPACK.

To test the structural adaptability of the model, the remaining 184 questionnaire data were used to conduct confirmatory factor analysis (CFA) on the 22 retained item data by using AMOS24.0 software. Following the approach of Hu and Bentler (1999) and Hair et al. (2010), χ^2^, CMIN/DF (χ^2^/df), RMSEA, SRMR, TLI, and CFI were selected as the core fitting indices. As shown in Table 3, the model running results showed that the fitting indices reached a good or acceptable level (χ^2^ = 422.389, *p* < 0.001; χ^2^/df = 2.081; RMSEA = 0.077; SRMR = 0.070; TLI = 0.897; CFI = 0.910). The final structural equation model (SEM) of TPACK perceived by preservice mathematics teachers is shown in Figure 2.

**Table 3:** PMT-TPACK structural equation modeling (SEM) fitting indicators.

Model	$\chi^{2}$	$\chi^{2}$ /df	RMSEA	SRMR	TLI	CFI
Suggested	Significant *p*-values expected	1–3	<0.08	<0.1	>0.90	>0.90
PMT-TPACK	422.389^***^	2.081	0.077	0.070	0.897	0.910
Result	Significant	Acceptable	Acceptable	Acceptable	Acceptable	Acceptable

^***^p < 0.001.

**Figure 2:**
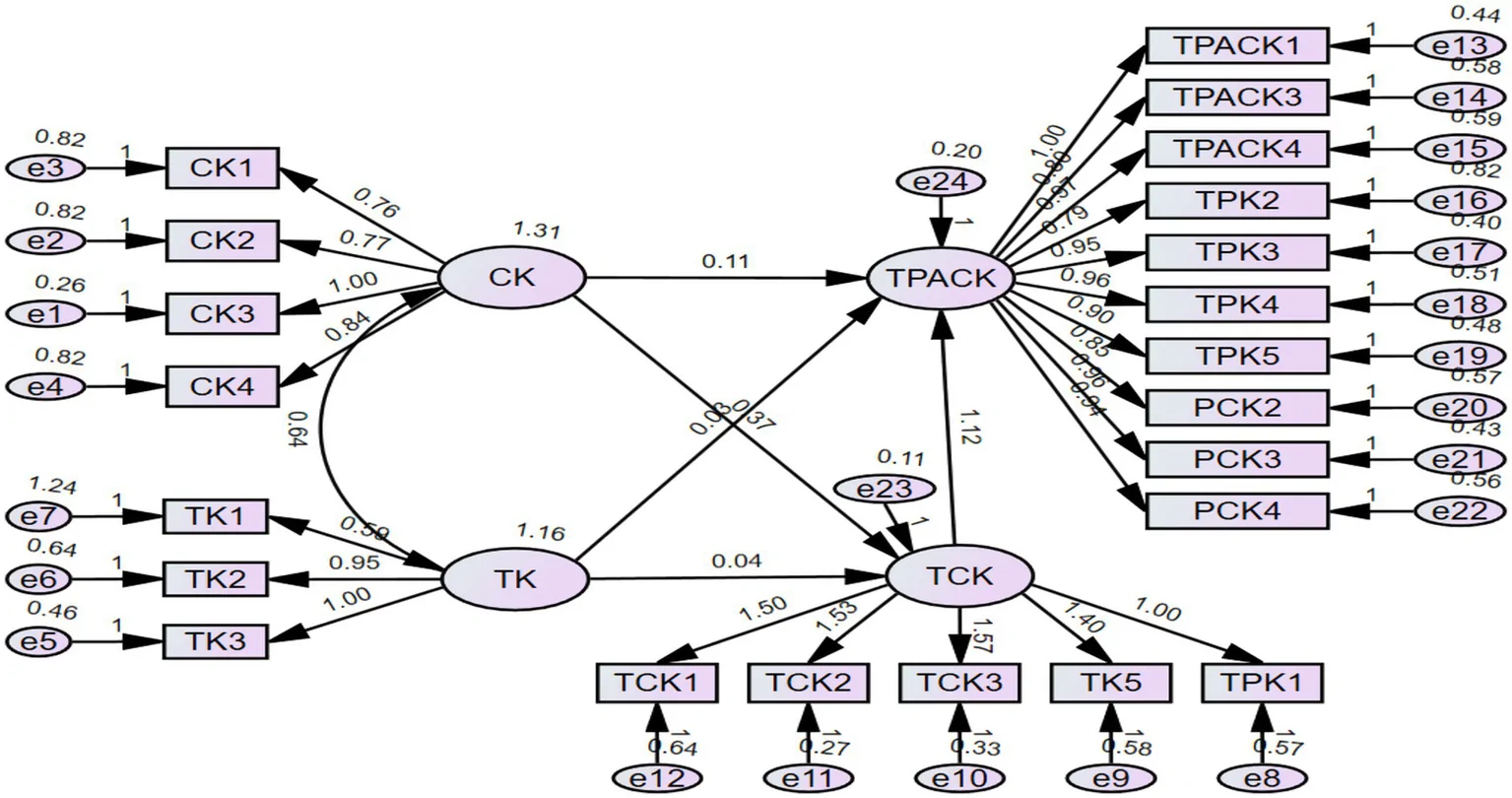
Path diagram of PMT-TPACK structural equation model (SEM).

Table 4 presents the path test results of the structural equation model for pre-service mathematics teachers’ PMT-TPACK: On one hand, two core paths (H1 and H5) were significantly supported. Content Knowledge (CK) had a significant positive effect on “Technological Content Knowledge (TCK)” (path coefficient 0.38, *p* < 0.001), indicating that solid mathematical knowledge serves as the foundation for teachers to present subject content with the aid of technology. The positive effect of TCK on TPACK comprehensive ability is stronger (path coefficient 1.12, *p* < 0.001), confirming that “the integrative ability of technological-mathematical knowledge” serves as the core mediator for preservice mathematics teachers in synthesizing technology, content, and pedagogy into practical teaching competence. On the other hand, H2, H3, and H4 were not supported by data, indicating that the direct effects of mathematical content knowledge (CK) on TPACK, technological knowledge (TK) on TCK, and TPACK itself were not significant. This suggests that pure mathematics knowledge or technological operational skills cannot be directly transformed into integrated teaching competencies, but must be achieved indirectly through the “technology-mathematics integration dimension” of TCK. This result suggests that the training of preservice mathematics teachers needs to strengthen the scenario based integration training of technical tools and mathematical content (such as using dynamic software to demonstrate the rules of function changes), rather than teaching subject knowledge or technical tools in isolation.

**Table 4:** Assumptions and path coefficients of PMT-TPACK structural equation model (SEM).

Hypothesis	Estimate	S.E.	C.R.	Result
*H1*: CK has a significant positive effect on TCK	0.38	0.05	6.97^***^	Yes
*H2*: CK has a significant positive impact on TPACK	0.12	0.08	1.43	No
*H3*: TK has a significant positive impact on TCK	0.04	0.04	1.09	No
*H4*: TK has a significant positive impact on TPACK	0.03	0.05	0.58	No
*H5*: TCK has a significant positive impact on TPACK	1.12	0.20	5.56^***^	Yes

^***^p < 0.001.

### Convergent validity

Following the approach of Hu and Bentler (1999) and Hair et al. (2010), the study validated the convergence validity of the structural model by testing composite reliability and extracting mean variance. Among them, CR is used to measure the internal consistency reliability of all items in the same dimension, and the higher its value, the stronger the correlation between each item. The acceptance threshold for CR in factor analysis is 0.6, and the ideal range is between 0.70 and 0.95. AVE measures the average degree of explanatory factors for all items in the same dimension, with an acceptance threshold of 0.40, indicating that each item can explain at least 40% of the corresponding factors on average.

The standardized factor loadings for each item in Figure 2 are shown in Table 5. Based on these loadings, calculate the CR and AVE values for the four TPACK core factors. The results showed that the CR values of all factors were higher than 0.6, specifically CK (0.77), TK (0.66), TCK (0.86), and TPACK (0.91). Except for TK, all factors met the ideal interval of 0.70–0.95, indicating good internal consistency reliability of each factor. The AVE values of all factors reach the critical threshold of 0.40, which is CK (0.46), TK (0.40), TCK (0.55), and TPACK (0.51), indicating that each item can explain more than 40% of the variance of its measurement indicators. Overall, the CR and AVE values of the four TPACK factors meet academic research standards, confirming the reliable convergence validity of the structural equation model constructed in this study.

**Table 5:** SEM standardization factor loading, CR, and AVE.

Latent variable	Item	Standardization factor load	CR	AVE
CK	CK1	0.69	0.77	0.46
	CK2	0.70		
	CK3	0.91		
	CK4	0.73		
TK	TK1	0.50	0.66	0.40
	TK2	0.79		
	TK3	0.85		
TCK	TCK1	0.73	0.86	0.55
	TCK2	0.86		
	TCK3	0.84		
	TK5	0.72		
	TPK1	0.60		
TPACK	TPACK1	0.80	0.91	0.51
	TPACK3	0.68		
	TPACK4	0.74		
	TPK2	0.61		
	TPK3	0.80		
	TPK4	0.77		
	TPK5	0.76		
	PCK2	0.70		
	PCK3	0.79		
	PCK4	0.74		

Correlations between demographic variables and TPACK.

Table 6 presents the demographic variables of the participants in this study, and the specific results are as follows: in terms of gender, males account for 49.46% and females account for 50.54%, with a sample structure close to equilibrium. In terms of grade distribution, juniors have the highest proportion (41.58%, 153 students), followed by sophomores (37.23%, 137 students), with a combined proportion of nearly 80%. Freshman students account for 13.86% (51 people), while graduate students only account for 7.34% (27 people), indicating that the research subjects are mainly undergraduate students in the middle and upper grades. In terms of educational practice courses, the proportion of choosing two practical courses is the highest (39.13%), while 34.24% of participants did not choose practical courses. Only 7.34% of participants completed four practical courses, indicating a relatively low overall participation rate in practical courses. In terms of participation in technical courses, over 50% of participants have completed three courses on the application of modern educational technology, mathematical models and experiments, or the application of mathematical teaching software (such as GeoGebra and Geometry Sketchpad), while only 3.80% have not participated in technical courses, indicating a relatively high overall participation rate in technical courses in the sample.

**Table 6:** Descriptive analysis of demographic variables.

Demographic variables	Specific classification	Number	Percentage
Gender	Male	182	49.46%
	Female	186	50.54%
Grade	Freshman	51	13.86%
	Sophomore	137	37.23%
	Junior	153	41.58%
	Graduate	27	7.34%
Practical courses	0	126	34.24%
	1	36	9.78%
	2	144	39.13%
	3	35	9.51%
	4	27	7.34%
Technical courses	0	14	3.80%
	1	87	23.64%
	2	65	17.66%
	3	198	53.80%
	4	4	1.09%

Table 7 shows Pearson correlation analysis results between the preservice mathematics teachers’ perceived dimensions of TPACK and demographic variables. Most variables show a significant positive correlation with weak to moderate strength, which is consistent with the characteristic that educational variables are influenced by multiple factors. The specific results are as follows: there is a significant positive correlation between grade and all dimensions of TPACK (CK: *r* = 0.16^**^, TCK: *r* = 0.18^**^, TPACK: *r* = 0.10^*^), indicating that as the learning stage progresses, preservice teachers gradually accumulate subject knowledge, technology-mathematics integration ability, etc. Gender is only slightly significantly negatively correlated with subject knowledge (CK) at the *p* < 0.05 level (*r* = −0.12^*^), indicating that boys have more confidence in mathematics subject knowledge compared to girls. There is a significant weak positive correlation between educational practice courses and various dimensions of TPACK, with a relatively weak correlation with TCK (*r* = 0.20^**^), which inspires preservice mathematics educators should pay attention to the effect of teaching practice on the preservice mathematics teachers’ integration ability of technology and mathematics subject content. The significant weak positive correlation between mathematics & technology courses and TPACK dimensions is the highest among all variables (TPACK: *r* = 0.24^***^, TCK: *r* = 0.23^***^), indicating that preservice mathematics educators should pay attention to the targeted integration training of technical tools and mathematical content (such as using dynamic software to demonstrate the rules of function changes) is an effective path for integrating technology, content, and pedagogy for preservice mathematics teachers.

**Table 7:** Pearson correlation analysis between TPACK dimensions and demographic variables.

Variable	Grade	Gender	EPC	TTC
CK	0.16^**^	−0.12^*^	0.15^**^	0.19^**^
TK	0.15^**^	0.10	0.11^*^	0.15^**^
TCK	0.18^**^	0.01	0.20^**^	0.23^**^
TPACK	0.10^*^	−0.07	0.13^*^	0.24^**^

^**^p < 0.01, ^*^p < 0.05.

## Conclusions and discussion

This study is based on questionnaire survey of 368 preservice mathematics teachers, using exploratory factor analysis and structural equation modeling to construct and validate a four-dimensional TPACK structural model, including content knowledge (CK), technological knowledge (TK), technological content knowledge (TCK), and integrated technological pedagogical content knowledge (TPACK), excluding PK, TPK and PCK. Among them, the items of TPK, PCK, and TPACK are merged into a single dimension of “technological pedagogical content knowledge (TPACK)”, indicating that participants have difficulty distinguishing these three types of highly integrated knowledge clearly. The results indicate that the model has good reliability and validity, and can stably reflect the cognitive structure of preservice mathematics teachers’ integrating technology into subject teaching. This study has also found that preservice teachers have the strongest confidence in TCK, which means they are relatively confident in applying technology to teach mathematics content. However, the confidence in TK is significantly low, especially in terms of self-evaluation in learning new technologies and using professional software. This result is similar to the study by Tondeur et al. (2016) among language teachers, indicating that preservice teachers generally feel a lack of confidence in the face of constantly evolving technology.

More importantly, TCK plays a crucial mediating role in the formation of TPACK. Both content knowledge and technological knowledge need to be effectively transformed into integrated technological subject teaching competence through the TCK “fusion link.” Merely mastering mathematical knowledge or technical operations is not sufficient to support high-quality technology integrated subject teaching. In addition, the level of participation in technical courses and educational practice courses has a significant weak positive impact on all dimensions of TPACK, with the role of technical courses being more prominent. The higher the grade, the higher the TPACK level, reflecting the cumulative effect of professional learning. The study also found that preservice mathematics teachers have certain difficulties in distinguishing TPK (technological pedagogical knowledge) from other composite dimensions. For example, in factor analysis, items originally belonging to TPK, such as “using internet search engines to prepare teaching materials”, are classified into TCK or TPACK dimensions, reflecting that they are more inclined to regard technology as a tool to support teaching content or overall teaching, and have not yet formed the awareness of independent combination of technology and pedagogy. In other words, they are more concerned with ‘what to teach with technology’ rather than ‘how to improve teaching methods with technology’. As Tondeur et al. (2012) pointed out, pre-service teacher education should not only focus on how to use technology, but also how technology can be used for teaching and learning.

The development of TPACK for preservice teachers is not achieved overnight, but gradually formed through curriculum learning, teaching practice, and continuous reflection. This also reminds us that preservice teacher training should focus on organically connecting learning, application, and reflection, providing systematic and coherent support for the development of abilities of integrating technology. As Koehler et al. (2007) proposed, developing TPACK is an iterative process that involves developing a deep understanding of the complex interactive relationships among content, pedagogy, technology, and their operating environments. Niess et al. (2009) proposed that when learning to integrate a specific technology into mathematics teaching, teachers can progress through five stages of development: cognition, acceptance, adaptation, exploration, and improvement. Kinuthia et al. (2010) also pointed out that TPACK carries various forms and variables in practice, and needs to be further developed through iterative cycles of application and reflection. Wu et al. (2012) also pointed out that actively participating in the design and implementation of technology integrated courses is a very promising strategy for teachers’ TPACK development.

Finally, we proposes the following suggestions. Firstly, technology application tasks should be appropriately integrated into teaching of mathematics courses in universities, so that preservice teachers can naturally come into contact with and practice using relevant technologies in the process of teaching mathematics. Secondly, university teacher educators should design a gradual and progressive training path from shallow to deep, gradually enhancing the preservice mathematics teachers’ technical application confidence and teaching transformation ability through micro teaching, lesson example discussions, and other methods. As Huang et al. (2022) observed that most teachers tended to develop a sense of competence and comfort as their understanding of TPACK deepened. However, over time, participants reported increased confidence and competence, suggesting that deliberate practice within supportive professional development contexts can mitigate these challenges. Thirdly, university teacher educators should clarify the specific requirements for technology integration in practical activities such as educational internships, and provide targeted guidance and feedback. Fourthly, for individuals with weak technical confidence, personalized support can be provided through specialized workshops, peer assistance, or mentorship to promote the balanced development of their technical integration abilities. As Alabdulaziz (2021) pointed out, if teachers think or perceive that it is uncomplicated and simple to use the digital technology, then they are willing and intent to spend more effort and time to learn how to do so, which would undoubtedly improve their performance. In contrast, if the digital technology is complicated and difficult to operate and use, then teachers would be unwilling to try to use it.

### Limitations and future research

This study has some limitations and needs to be improved in future. Firstly, the sample was from school of mathematics and physics at a local comprehensive university in China, and the geographical and school types are relatively single. Whether the results are applicable to normal students in other regions still needs to be verified. In addition, a subset of 184 data points was utilized to conduct the confirmatory factor analysis on the 22 retained items. While the methodological practice of splitting the sample to conduct independent exploratory and confirmatory factor analyses is appropriate, a sample size of 184 for a structural model containing 22 observation items resides on the lower boundary of statistical acceptability. This specific sample-to-item ratio risks parameter instability and increases the margin of error within the final model. Secondly, this study used a cross-sectional survey, which can only reflect the situation at a certain time point and is difficult to see the changes in TPACK ability of preservice mathematics teachers during the learning process. In the future, tracking design can be adopted to observe the development process of preservice mathematics teachers from enrollment to graduation. Thirdly, the data mainly relies on self-assessment of the questionnaire. A possible drawback of this design is that the participants could be responding to the questions based on how they think the survey administrators and teacher education program expect them to respond. This systematic bias could influence the participants’ responses, thereby influencing instrument validity and reliability. Subsequent research should combine classroom observation, teaching design analysis, reflective practice, or interviews with various methods to complement each other and gain a more authentic understanding of the development of preservice mathematics teachers’ TPACK. Therefore, TPACK pathways described still need to be further validated through both in-depth qualitative studies and an investigation of whether better fit could be derived from alternative modeling of the structural relationships. Finally, regarding that the exclusion of PK, TPK, and PCK, the absence of significant pathways between CK and TPACK, between TK and TCK, as well as the integration of TPK, PCK, and TPACK into a single dimension of “technological pedagogical content knowledge” (TPACK), TPACK need to be considered with respect to the design of its survey items. Further studies are needed to examine the specified relationships in this pathway.

These limitations provide scope for further study into this area. Firstly, there is a need to make clearer references to mathematics in the TPACK items. The pathways between various knowledge components in TPACK, TPACK need to be re-examined with these revised survey items. In addition to TPACK items, there is also a need to consider if adding the item “Without using technology” into PK and CK items could better dissociate it from TK items. The references to mathematics in the CK items also need to be reconsidered for clearer interpretation of CK items and PK items. The structural equation modeling needs to be repeated with these survey items to determine the extent to which the design of these survey items could have impacted the study results. Secondly, there is a need to validate the results of this study through a national study. Cluster sampling could be used to ensure adequate representation by either preservice mathematics teacher profile or school profile. This can provide validation of the results of this study. In addition, this study can be replicated with preservice mathematics teachers to determine if their perceived TPACK pathways are similar. Another area of future research is to validate the TPACK pathways through qualitative studies of preservice mathematics teachers across time through classroom observations, in-depth interviews, and the examination of their ICT lesson designs. Such kinds of in-depth studies also shed light on the presence of TPACK formation pathways that have not been found in this study. This would shed light on the epistemological nature of TPACK. This will provide insights about the development of these pathways across time. It can be used to assess if strategies used in preservice teacher education courses are effective for facilitating preservice teachers’ TPACK development. It can also be used to develop instructional models for facilitating the content-centered pathway of TPACK formation.

## Data Availability

The original contributions presented in the study are included in the article/Supplementary material, further inquiries can be directed to the corresponding author.
