# Supplementary material for: Examining preservice mathematics teachers’ perceptions of technological pedagogical content knowledge (TPACK) pathways: a structural equation modeling approach
Source: Front Psychol. 2026 Oct 8;17:1929322. doi: 10.3389/fpsyg.2026.1929322 (PMC13689567; doi:10.3389/fpsyg.2026.1929322)
Supplement: Supplementary file 1 [file Supplementary_file_1.doc]

**Appendix**

Pre-service mathematics teachers’ TPACK

Directions for students:

This questionnaire is to understand pre-service mathematics teachers' perspectives on pedagogical content knowledge for integrating technology into mathematics instruction. The questionnaire consists of 33 questions, each with seven options: "Strongly disagree, disagree, somewhat disagree, uncertain, somewhat agree, agree, strongly agree." Carefully read each question and select the option that best aligns with your actual situation. There are no `right’ or `wrong’ answers. Your opinion is what is wanted. Be sure to give an answer for all questions. If you change your mind about an answer, just cross it out and circle another. Some statements in this questionnaire are fairly similar to other statements. Don’t worry about this. Simply give your opinion about all statements. Thank you very much for your cooperation.

strongly agree

agree

somewhat agree

uncertain

somewhat disagree

disagree

Strongly disagree

1.I can easily learn new technologies	1---2---3---4---5---6---7

2.I can use basic image software (such as Meitu Xiuxiu) 1---2---3---4---5---6---7

3.I can use basic video software (such as Jianying)	1---2---3---4---5---6---7

4.I have sufficient understanding of mathematical knowledge, such as mathematical concepts

1---2---3---4---5---6---7

5.I have confidence in becoming a math teacher	1---2---3---4---5---6---7

6.I can use software such as Excel for data analysis	1---2---3---4---5---6---7

7.I can use various methods to deepen my understanding of mathematical knowledge

1---2---3---4---5---6---7

8.I can keep up with the latest developments in mathematics teaching and think about related issues

1---2---3---4---5---6---7

9.I can effectively organize and manage the classroom	1---2---3---4---5---6---7

10.I can adopt different teaching styles based on the characteristics of students

1---2---3---4---5---6---7

11.I can use appropriate tools (such as Tencent Meeting) for themed teaching and promote students' online learning 1---2---3---4---5---6---7

12.I can use various methods to monitor students' learning performance 1---2---3---4---5---6---7

13.I can use basic office applications such as PPT	1---2---3---4---5---6---7

14.When not using technology, I can facilitate meaningful discussions among students on the topics they are learning 1---2---3---4---5---6---7

15.I can expand students' thinking by setting challenging tasks for them 1---2---3---4---5---6---7

16.I can use software specifically designed for mathematics teaching or learning, such as a geometry sketchpad

1---2---3---4---5---6—-7

17.I can use various teaching methods in the classroom	1---2---3---4---5---6---7

18.I can use appropriate technology (such as electronic whiteboards) to present math teaching content

1---2---3---4---5---6---7

19.I can use appropriate technology to promote students' mathematics learning

1---2---3---4---5---6---7

20.I can independently solve technical problems when using technology 1---2---3---4---5---6---7

21.I can use some technologies (such as databases) to conduct research on mathematics teaching

1---2---3---4---5---6---7

22.I can choose appropriate techniques to enhance my teaching skills 1---2---3---4---5---6---7

23.When not using technology, I can prepare course plans 1---2---3---4---5---6---7

24.I can use Internet search engines (such as Baidu) to prepare teaching materials

1---2---3---4---5---6---7

25.I can teach mathematics on online platforms	1---2---3---4---5---6---7

26.I can promote collaboration among students by using technology, such as online teaching platforms

1---2---3---4---5---6---7

27.I can think about how to use technology to teach mathematics in online teaching

1---2---3---4---5---6---7

28.I can use appropriate technology (such as electronic whiteboards) to infiltrate mathematical culture and ideas

1---2---3---4---5---6---7

29.When not using technology, I can choose appropriate and effective math teaching strategies

1---2---3---4---5---6---7

30.I can choose the appropriate technology to improve the teaching method of a class

1---2---3---4---5---6---7

31.When not using technology, I can solve common problems that students encounter in mathematics learning

1---2---3---4---5---6---7

32.I can design student-centered activities that combine mathematical content, pedagogy and technology

1---2---3---4---5---6---7

33.I can design web-based assignments (such as Study Pass) for mathematics learning

1---2---3---4---5---6---7

Your grade:_____(1.Freshman	2.Sophomore	3.Junior	4.Master's student)

Gender:______(1. Male	2. Female)

Education practice courses (multiple choice):________(1.Teaching practicum 2.Microteaching

3.Educational probation	4.Educational Research)

Teaching technology courses (multiple choice):________(1.Application of modern educational technology 2.Geometry sketchpad 3.Mathematical models and experiments 4.Other________)
